# Six-Year Changes in Myopic Macular Degeneration in Adults of the Singapore Epidemiology of Eye Diseases Study

**DOI:** 10.1167/iovs.61.4.14

**Published:** 2020-04-16

**Authors:** Yee-Ling Wong, Charumathi Sabanayagam, Chee-Wai Wong, Yin-Bun Cheung, Ryan Eyn Kidd Man, Anna Chwee-Hong Yeo, Gemmy Cheung, Audrey Chia, Anthony Kuo, Marcus Ang, Kyoko Ohno-Matsui, Tien-Yin Wong, Jie Jin Wang, Ching-Yu Cheng, Quan V. Hoang, Ecosse Lamoureux, Seang-Mei Saw

**Affiliations:** 1 Singapore Eye Research Institute, Singapore National Eye Centre, Singapore; 2 Saw Swee Hock School of Public Health, National University of Singapore, Singapore; 3 R&D Vision Sciences AMERA, Essilor International, Singapore; 4 Duke-NUS Medical School, Singapore; 5 Yong Loo Lin School of Medicine, National University of Singapore and National University Health System, Singapore; 6 Department of Ophthalmology, Duke University Medical Center, Durham, North Carolina, United States; 7 Department of Biomedical Engineering, Duke University, Durham, North Carolina, United States; 8 Department of Ophthalmology and Visual Science, Tokyo Medical and Dental University, Tokyo, Japan; 9 Department of Ophthalmology, Harkness Eye Research Institute, Columbia University College of Physicians and Surgeons, New York, New York, United States

**Keywords:** epidemiology, myopic macular degeneration, pathologic myopia, vision-related quality of life, myopia

## Abstract

**Purpose:**

To examine the 6-year incidence, progression, associated risk factors, and impact of myopic macular degeneration (MMD) in a myopic population in Singapore.

**Methods:**

We examined myopic (spherical equivalent ≤–0.5 diopters) adults (*N* = 2157 persons and 3661 eyes) who were phakic at baseline and participated in both baseline and 6-year follow-up visits of the Singapore Epidemiology of Eye Diseases study. Eye examinations, including visual acuity, subjective refraction and axial length (AL) measurements, were performed. MMD was graded from fundus photographs following the META-PM classification. Vision-related quality of life was assessed with Rasch-transformed scores from reading, mobility, and emotional domains of the Impact of Vision Impairment questionnaire.

**Results:**

The 6-year eye-specific incidence of MMD among myopic eyes was 1.2% (95% CI, 0.9%–1.6%). Older age, worse spherical equivalent, and longer AL at baseline were associated with MMD incidence (all *P* < 0.001). The 6-year eye-specific progression of MMD in 288 eyes with baseline MMD was 17.0% (95% CI, 12.6%–21.4%). More severe MMD at baseline, worse spherical equivalent, and longer AL (all *P* < 0.05) were associated with MMD progression. Patients with Meta-PM categories 3 and 4 had worse best-corrected visual acuity and poorer vision-related quality of life outcomes than those without MMD (all *P* < 0.05).

**Conclusions:**

Over a 6-year period, one in 80 myopic eyes developed MMD and one in six with existing MMD had MMD progression. Myopia severity and AL were strong predictors of MMD development and progression. Eyes with severe MMD were at higher risk of MMD progression than those with less severe MMD, and were associated with poorer visual acuity and vision-related quality of life.

Myopia is a major public health issue and is linked to the development of vision-threatening complications, such as myopic macular degeneration (MMD), retinal detachment and cataract.^1^^,^^2^ MMD can result in permanent visual impairment,^2^^–^^4^ and its prevalence ranges from 0.2% to 3.8% among general adult populations in India, Australia, China, Taiwan, Japan, and Singapore.^3^^,^^5^^–^^13^

Most studies reporting on the longitudinal changes in MMD have focused on the progression pattern of MMD in eyes with preexisting MMD,^6^^,^^7^ with very limited evidence on the incidence of MMD,^14^ except for two studies conducted in Chinese adult populations.^15^^,^^16^ A low 5-year eye-specific incidence of MMD (0.2%) among 2236 myopic eyes of adults living in rural China was reported.^15^ Another study reported a 10-year incidence of MMD at 19.0% in 79 highly myopic eyes of Chinese adults.^16^ However, this study reported MMD changes among highly myopic adults only and did not consider individuals with low or moderate myopia who are also at risk of developing MMD.^11^

In population-based studies, the 5-year progression rate of MMD ranged from 15.1% to 35.3%,^6^^,^^7^^,^^15^ and the 10-year progression rate was reported to be 77.4% of eyes with MMD.^16^ In contrast, clinic-based case series among Japanese patients reported high progression rates of MMD (54.7% to 74.3%) in very highly myopic eyes of patients with mean follow-up of 10 years or more.^17^^,^^18^ Most studies on the progression pattern of MMD have been limited to ethnically homogenous populations.

In this study, we investigated the eye-specific 6-year cumulative incidence, progression, associated risk factors and impact of MMD among Chinese, Indians, and Malays myopic adults who participated in the population-based Singapore Epidemiology of Eye Diseases (SEED) cohort study.

## Methods

### Study Population

The SEED study comprises three population-based cohort studies of adults aged 40 to 80 years in Singapore, with the baseline studies conducted from 2004 to 2011 (the Singapore Malay Eye Study [2004–2006], the Singapore Indian Eye Study [2007–2009], and the Singapore Chinese Eye Study [2009–2011]) and the 6-year follow-up studies from 2010 to 2017 (Singapore Malay Eye Study 2 [2010–2013], Singapore Indian Eye Study 2 [2013–2015], and Singapore Chinese Eye Study 2 [2015–2017]). The detailed study methodologies have been described elsewhere.^19^^–^^21^ Briefly, 10,033 subjects (3280 Malays, 3400 Indians, and 3353 Chinese) participated in the baseline examination (total response rate of 75.6%). After 6 years, 1449 subjects (14.4%) were ineligible to participate in the follow-up examination owing to the development of cognitive and/or severe mobility impairments, migration to other countries, imprisonment, or having died during the 6-year period. Of the remaining 8584 eligible subjects, 6762 (78.8%) participated in the 6-year follow-up examination (Singapore Malay Eye Study 2, Singapore Indian Eye Study 2, and Singapore Chinese Eye Study 2). The study adhered to the Declaration of Helsinki, and ethics approval was obtained from the Singapore Eye Research Institute Institutional Review Board.

### Inclusion and Exclusion Criteria

Of the 10,033 subjects at baseline, we first excluded 1317 with the following conditions: (1) procedures that affected the native refractive state of the eye, such as a history of cataract surgery resulting in aphakia or pseudophakia, and/or self-reported refractive surgery in both eyes; (2) missing refraction data in both eyes; (3) combination of cataract surgery in one eye and missing refraction data in the other eye; and (4) missing or ungradable fundus photographs.^11^ Of the remaining 8716 patients, we included only those with myopia at baseline (*n* = 3108) because only myopes are at risk of MMD development.^11^ At the 6-year follow-up examination, 2185 of the 3108 myopic subjects (70.3%) were reexamined. Of the 2185 reexamined subjects, 2157 (98.7%) had gradable fundus photographs to evaluate for MMD at both baseline and follow-up visits, and were included in this analysis.

To examine the longitudinal changes in MMD among those with previous cataract surgery at baseline, we performed a separate additional analysis on eyes that had undergone cataract surgery. The eyes with previous cataract surgery were not included in the main analyses, because the measurements taken during refraction are not truly reflective of their refractive error. Of 682 persons with cataract surgery, 536 (78.6%) had gradable fundus photographs at both baseline and follow-up visits and were analyzed separately.

### Visual Acuity Assessment

The monocular presenting visual acuity (PVA) was measured using the logMAR chart (Lighthouse International, New York, NY) at 4 m with the participants wearing their habitual correction. If the largest number could not be read at 4 m, the chart was moved closer to the participant (2 m), then counting fingers, hand motion, or light perception was assessed in that order. Subjective refraction and best-corrected VA (BCVA) measurements were conducted on the same day by a trained optometrist.

### Refraction, Biometry Measurements, and Ocular Examination

Autorefraction was performed using an auto-refractometer (model RK5; Canon, Inc., Ltd., Tochigiken, Japan). Refraction was then subjectively refined by the study optometrists until the BCVA was obtained. The results from subjective refraction were used in the analysis. The spherical equivalent (SE) of the refractive error was defined as sphere plus half cylinder. Myopia was defined as an SE of –0.5 diopters (D) or less in at least one eye. Low, moderate, and high myopia were defined as an SE of between –3.0 D and –0.5 D, between –5.0 D and –3.0 D, and –5.0 D or less, respectively. Axial length (AL) was measured using noncontact partial coherence interferometry (IOL Master V3.01; Carl Zeiss Meditec AG, Jena, Germany).

### Fundus Photography and Grading

After cycloplegia, color fundus photographs of Early Treatment Diabetic Retinopathy Study standard field 1 (centered on the optic disc) and Early Treatment Diabetic Retinopathy Study standard field 2 (centered on the fovea) were captured for each eye using standardized settings with a nonmydriatic retinal camera (Canon CR-DGi with 10D SLR back; Canon, Inc., Tokyo, Japan). A detailed fundus photograph grading was performed by a single trained grader (W.Y.L.). The fundus photographs of both eyes were graded using a standardized protocol, and the grader was masked to the subjects’ characteristics. Adjudication was performed by retinal specialist (W.C.W.). Grading of MMD Meta-PM categories by the retinal specialist (W.C.W.) and the trained grader (W.Y.L.) was compared; the kappa statistics showed high inter-grader agreement (diagnosis of Meta-PM categories were 0.94 [between W.C.W. and W.Y.L.]).

### Definition of MMD

Based on the International META-PM Classification,^22^ the presence of MMD was defined and classified into the following categories: no macular lesions (category 0); tessellated fundus only (category 1); diffuse chorioretinal atrophy (category 2); patchy chorioretinal atrophy (category 3); and macular atrophy (category 4). Plus lesions, which supplemented the Meta-PM categories comprised lacquer cracks, choroidal neovascularization and Fuchs’ spot. Based on fundus photograph grading, an eye was considered to have MMD, if Meta-PM category 2, 3, 4, or any plus lesion, was observed.^3^ The presence of optic disc abnormalities (optic disc tilt, peripapillary atrophy [PPA] and peripapillary intrachoroidal cavitation) was also graded, although they are not part of the META-PM classification. Optic disc tilt was defined by an oval optic disc with a tilt ratio (minimum diameter to maximum diameter) of less than 0.75. PPA was defined using the classification by Curtin and Karlin.^23^ Peripapillary intrachoroidal cavitation was observed as an elevated, well-circumscribed, dome-shaped, yellow–orange lesion adjacent to the optic disc and PPA.^24^

### Definition of Incidence and Progression of MMD

The incidence of MMD was defined as the development of any MMD during the 6-year follow-up period in myopic eyes without MMD at baseline examination.

Progression of MMD was defined as an increase in Meta-PM category (change from Meta-PM category 2 during baseline to Meta-PM category 3 or 4 during follow-up, or change from Meta-PM category 3 during baseline to Meta-PM category 4 during follow-up), expansion of existing chorioretinal atrophy, development of a plus lesion, or enlargement of an existing plus lesion in eyes with preexisting MMD at the baseline examination. The expansion of existing chorioretinal atrophy or plus lesion was defined by an enlargement of the area of chorioretinal atrophy or plus lesion by more than 50%. The area of the chorioretinal atrophy was assessed by Image J software (https://imagej.nih.gov/ij/; provided in the public domain by the National Institutes of Health, Bethesda, MD).

### Risk Factor Assessment

Detailed questionnaires, administered by trained research staff through face-to-face interviews, were used to collect demographic data (age, sex, and ethnicity), socioeconomic characteristics (education level), and general medical history from participants in their preferred language (English or mother tongue). Education level was classified as primary/below education and secondary/above education.

### Other Eye Examinations

Other ocular diseases included cataract, glaucoma, diabetic retinopathy (DR) and AMD. The detailed study methodologies for the identification and grading of each of these ocular diseases in SEED have been described elsewhere.^25^^–^^29^ Cataract was defined using the Wisconsin Cataract Grading System.^30^ Any cataract was defined as the presence of any nuclear (grade ≥4), cortical (≥25% of total lens area), or posterior subcapsular (≥5% of total lens area) cataract.^29^ Glaucoma was graded according to the International Society of Geographical and Epidemiological Ophthalmology scheme.^27^^,^^28^ The grading for AMD was conducted using the Wisconsin Age-related Maculopathy grading system.^26^ Any AMD was defined as the presence of early AMD (soft indistinct or reticular drusen or both soft, distinct drusen plus retinal pigment epithelium abnormalities) or late AMD (either neovascular AMD or geographic atrophy). The grading for DR was performed using a modification of the Airlie House classification system for the Early Treatment Diabetic Retinopathy Study.^25^ Any DR was defined as the presence of microaneurysms, hemorrhages, cotton wool spots, intraretinal microvascular abnormalities, hard exudates, venous beading, and new vessels.

### Assessment of Vision-Related Quality of Life

At the 6-year follow-up visit, vision-related quality of life (VRQoL) was assessed using the 32-item Impact of Vision Impairment (IVI) questionnaire.^31^ The IVI gives an overall comprises three component scales: reading and accessing information (reading), mobility and independence (mobility), and emotional well-being (emotional).^32^ Rasch analysis was undertaken to assess the psychometric properties of the IVI in the current sample using the Andrich rating scale model ^33^ with Winsteps software (version 3.91.2, Chicago, IL). Rasch analysis is a form of item response theory where a person's questionnaire score is modeled as a logistic function of the difference between the person's ability level and the difficulty of the questions, with this score expressed as log of the odds units or logits.^34^

### Statistical Analysis

Baseline characteristics of the participants who were included and excluded for the analyses were compared using the Fisher exact test or *t*-test as appropriate for the variable. The eye-specific cumulative incidence and progression rates were calculated based on the development of MMD in eyes without preexisting MMD and on the progression of MMD in eyes with preexisting MMD, respectively. Risk factors of MMD incidence and progression, including demographic (age, sex, and ethnicity), socioeconomic (education), ocular (SE or AL), and ocular conditions (baseline disc lesions, cataract, glaucoma, DR, and AMD) variables were assessed and estimated as risk ratios (RR) using backwards stepwise multivariable modified Poisson regression models.^35^ To account for the correlation between the two eyes, generalized estimating equations were additionally incorporated within the regression models, and robust standard errors for clustered data were shown.

To examine the cross-sectional association between MMD and vision (BCVA and VRQoL) at the 6-year follow-up, participants with ocular comorbidities, including cataract, glaucoma, AMD, and DR, were excluded from this part of the analyses. The association between MMD and BCVA in the better-seeing eye was assessed by a multivariable-adjusted linear regression model adjusted for age, ethnicity, sex, education, and SE, whereas the association between MMD and each end point (VRQoL scores) was assessed using a multivariable-adjusted linear regression model adjusted for age, ethnicity, sex, education, SE, and PVA. The further adjustment for PVA in the better-seeing eye in the latter analysis was to account for potential confounding from uncorrected refractive errors. Additionally, MMD severity (no MMD, Meta-PM category 2, and Meta-PM category 3 or 4) was also analyzed as an ordinal variable against each end point. Statistical software Stata, version 13.1 (StataCorp LP, College Station, TX) was used for all analyses.

**Table 1.** Baseline Characteristics of Included Myopic Participants and Excluded Myopic Participants at the 6-Year Follow-up Examination of the SEED Study

	Included Participants	Excluded Participants
	With Myopia *(n* = 2157)	With Myopia (*n* = 951)
Age, mean (SD), y	54.2 (8.4)	59.2 (11.2)
Ethnicity, *n* (%)		
Chinese	1065 (49.4)	255 (26.8)
Malay	496 (23.0)	378 (39.8)
Indian	596 (27.6)	318 (33.4)
Sex, *n* (%)		
Female	1154 (53.5)	443 (46.6)
Male	1003 (46.5)	508 (53.4)
Education level, *n* (%)		
Primary/lower education	1359 (63.0)	381 (40.1)
Secondary/above education	798 (37.0)	570 (59.9)
SE, mean (SD), D*	–2.9 (2.8)	–2.6 (2.6)
AL, mean (SD), mm^*^	24.6 (1.5)	24.3 (1.3)
Baseline MMD, *n* (%)^*^		
Yes	202 (9.4)	148 (15.6)
No	1955 (90.6)	803 (84.4)
Baseline MMD meta-PM categories, *n* (%)^*^		
0	1068 (49.5)	439 (46.2)
1	887 (41.1)	364 (38.3)
2	190 (8.8)	144 (15.1)
3	11 (0.5)	4 (0.4)
4	1 (0.1)	0 (0.0)
Baseline plus lesions, *n* (%)^*^		
Yes	18 (0.8)	5 (0.5)
No	2139 (99.2)	946 (99.5)

SD, standard deviation.

* Data were derived from the worse eye (defined as eye with more severe MMD level, or the eye with higher myopia level in participants who had the same MMD category for both eyes).

## Results

Of the 3108 phakic subjects with myopia at baseline, 2157 eligible participants (69.4%) had gradable fundus photographs at both the baseline and 6-year follow-up visits. Participants who were ineligible, lost to follow-up, had missing or ungradable fundus photographs were older (*P* < 0.001), were more likely to be Malays (*P* < 0.001), were male (*P* < 0.001), had a higher level of education (*P* < 0.001), were less myopic (*P* = 0.004), had a shorter AL (*P* < 0.001), and were more likely to have MMD at baseline (*P* < 0.001), compared with the 2157 participants who were included in analyses (Table 1). A total of 3661 myopic eyes in 2157 participants were analyzed, comprising 3373 eyes without preexisting MMD in 2009 participants and 288 eyes with preexisting MMD in 202 participants.

**Table 2.** Development of MMD Lesions Over a 6-Year Period Among New MMD Cases in the SEED Study

	Eyes With Any New MMD Cases over a 6-Year Period (*n* = 42 eyes)
Development of new chorioretinal atrophy, *n* (%)	
Development of new diffuse chorioretinal atrophy	40 eyes (95.2)
Development of new patchy chorioretinal atrophy	2 eyes (4.8)
Development of new macular atrophy	0 eye (0.0)
Development of new plus lesions, *n* (%)	
Development of new lacquer crack(s)	1 eye (2.4)^*^
Development of new Fuchs’ spot/CNV	0 eye (0.0)

CNV, choroidal neovascularization.

* The same eye with development of new lacquer cracks had development of new diffuse chorioretinal atrophy.

### Incidence of MMD in Eyes With Myopia

**Figure 1.**
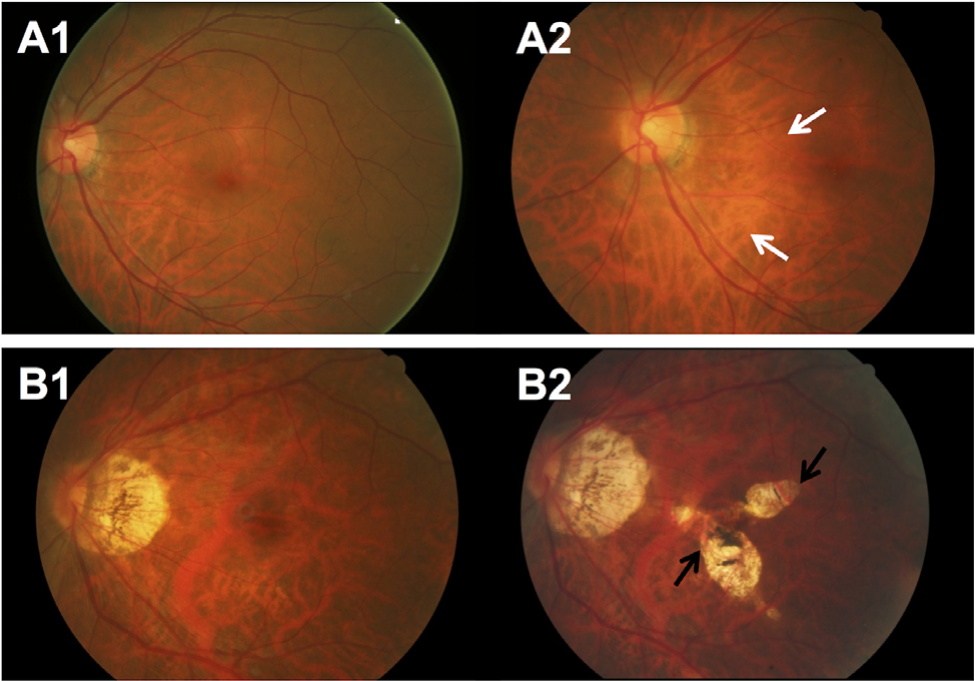
Fundus photographs of eyes with development of new MMD over a 6-year period. (**A**) Fundus photographs from a 48-year-old Malay woman showing development of incident MMD (development of diffuse chorioretinal atrophy) over a follow-up of 6 years. (**A1**) Image from the left eye obtained at baseline visit (SE of –6.9 D and AL of 27.7 mm) showing fundus tessellation only. (**A2**) Six years later, diffuse chorioretinal atrophy (*white arrows*) developed. (**B**) Fundus photographs from a 64-year-old Chinese woman showing development of incident MMD (development of patchy chorioretinal atrophy) over a follow-up of 6 years. (**B1**) Image from the left eye obtained at baseline visit (SE of –10.3 D and AL of 27.8 mm) showing fundus tessellation only. (**B2**) Six years later, several well-defined patchy chorioretinal atrophies (*black arrows*) in the macular region and choroidal neovascularization (CNV) developed at the fovea.

The 6-year cumulative incidence of MMD was 1.2% (95% confidence interval [CI], 0.9%–1.6%) of 3373 myopic eyes without MMD at baseline in the SEED study, with a higher incidence of 2.1% (95% CI, 0.8%–3.4%) among 478 highly myopic eyes without MMD at baseline. Of the 42 myopic eyes with newly developed MMD, 40 (95.2%), 2 (4.8%), and 1 (2.4%) had incident diffuse chorioretinal atrophy, patchy chorioretinal atrophy, and lacquer cracks, respectively (Table 2). All 42 eyes with MMD development at 6 years had tessellated fundus at baseline. Most eyes (97.6%) with MMD development at 6 years had presence of PPA at baseline. Figure 1 shows the baseline and follow-up fundus photographs of eyes that had incident MMD over the follow-up period. The incidence of MMD was higher in eyes with any cataract at baseline compared to those without (2.7% vs. 1.0%; *P* = 0.006). Particularly, the incidence of MMD was significantly higher in eyes with nuclear cataract at baseline compared to those without (3.5% vs. 1.1%; *P* = 0.007), but was not significantly higher in those with cortical cataract at baseline compared with those without cortical cataract (2.2% vs. 1.1%; *P* = 0.08).

In multivariable regression models, the risk of MMD development increased significantly with older age (RR of 1.12 per year increase; *P* < 0.001), greater myopic SE (RR of 1.3 per 1 D decrease; *P* < 0.001), and longer AL (RR of 1.7 per 1 mm increase; *P* < 0.001; Table 3). The presence of any cataract at baseline was not an independent risk factor for MMD development. The incidence of MMD was not significantly different with presence of baseline glaucoma, AMD, and DR (all *P* > 0.05).

**Table 3.** The 6-Year Incidence of MMD Among Myopic Eyes at Risk of MMD Development in the SEED Study (*n* = 2009; 3373 Eyes at Risk)

			Six-Year					
			Eye-Specific		Unadjusted		Multivariate-	
			Incidence of MMD		RRs		Adjusted RRs	
	No. at							
	Risk	*n*	%	95% CI	RR (95% CI)	*P* Value	RR (95% CI)	*P* Value
Cataract at baseline								
Yes	563	15	2.7	1.3–4.0	2.7 (1.3–5.5)	0.006	0.8 (0.3–2.1)^*^	0.65
No	2,742	27	1.0	0.6–1.4	Reference		Reference	
Age group at baseline, year								
40–49	1,466	6	0.4	0.1–0.7				
50–59	1,266	16	1.3	0.6–1.9				
60–69	508	14	2.8	1.3–4.2				
≥70	133	6	4.5	0.9–8.1				
*P* value for trend				<0.001				
Age at baseline, per 1 year	3,373	42	–	–	1.1 (1.1–1.1)	<0.001	1.1 (1.1–1.2)^*^	<0.001
Ethnicity								
Indian	961	9	0.9	0.3–1.5	Reference		Reference	
Chinese	1,681	22	1.3	0.8–1.9	1.4 (0.6–3.5)	0.47	0.9 (0.4–2.5)^*^	0.91
Malay	731	11	1.5	0.6–2.4	1.6 (0.6–4.5)	0.37	1.7 (0.6–5.0)^*^	0.30
Sex								
Male	1532	17	1.1	0.6–1.6	Reference		Reference	
Female	1841	25	1.4	0.8–1.9	1.2 (0.6–2.5)	0.57	1.5 (0.7–2.9)^*^	0.28
Myopia levels at baseline								
Low myopia (SE between –0.5 and –2.99 D)	2,251	20	0.9	0.5–1.3				
Moderate myopia (SE between –3.0 and –4.99 D)	644	12	1.9	0.8–2.9				
High myopia (SE worse than –5.0 D)	478	10	2.1	0.8–3.4				
*P* value for trend				0.02				
SE at baseline, per 1 D	3,373	42	–	–	1.2 (1.1–1.3)	0.001	1.3 (1.1–1.4)^*^	<0.001
AL at baseline, mm								
<26.5 mm	3,103	32	1.0	0.7–1.4				
≥26.5 mm	270	10	3.7	1.4–6.0				
								
AL at baseline, per 1 mm	3,373	42	–	–	1.5 (1.1–1.9)	0.004	1.7 (1.3–2.3)^†^	<0.001
Education level								
Primary/lower education	1,127	15	1.3	0.7–2.0	0.9 (0.4–1.8)	0.78	1.0 (0.4–2.5)^*^	0.97
Secondary/above education	2,246	27	1.2	0.8–1.7	Reference		Reference	
Optic disc tilt at baseline								
Yes	688	14	2.0	1.0–3.1	2.0 (0.9–4.0)	0.07		
No	2,685	28	1.0	0.7–1.4	Reference			

* Multivariable modified Poisson regression models to obtain adjusted RRs were adjusted for age, sex, ethnicity, education level, and SE and for correlation between eyes in each individual.

† Multivariable modified Poisson regression models to obtain adjusted RRs were adjusted for age, sex, ethnicity, education level, and AL and for correlation between eyes in each individual.

### Progression of MMD

**Figure 2.**
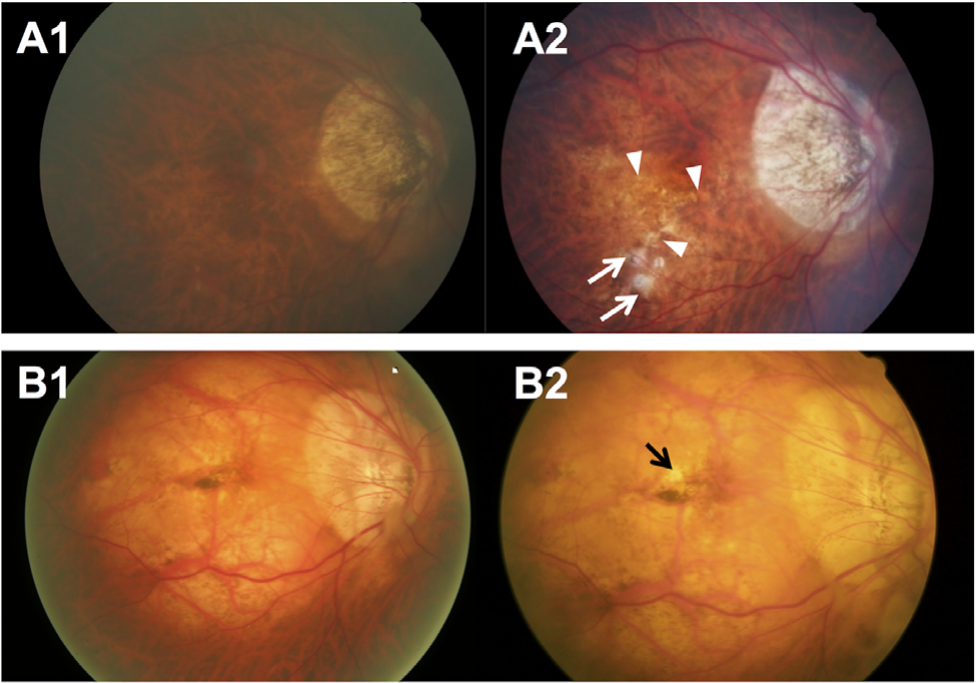
Fundus photographs of eyes showing MMD progression (from Meta-PM category 2 at baseline to Meta-PM category 3 at follow-up) with development of new patchy chorioretinal atrophy over a 6-year period. (**A**) Fundus photographs from a 62-year-old Chinese woman showing MMD progression with development of new patchy chorioretinal atrophy over a follow-up of 6 years. (**A1**) Image from the right eye obtained at baseline visit (SE of –2.8 D and AL of 28.4 mm) showing diffuse chorioretinal atrophy on the inferior–temporal region of the macula. (**A2**) Six years later, several spotted patchy chorioretinal atrophies (*white arrows*) and lacquer cracks (*white arrowheads*) developed, and the preexisting diffuse chorioretinal atrophy expanded. Enlargement of PPA was observed as well. (**B**) Fundus photographs from a 50-year-old Malay man showing MMD progression with development of new patchy chorioretinal atrophy over a follow-up of 6 years. (**B1**) Image from the right eye obtained at baseline visit (SE of –12.3 D) showing diffuse chorioretinal atrophy on the entire macular region and Fuchs’ spot at the fovea. (**B2**) Six years later, a small new patchy chorioretinal atrophy (*black arrow*) developed in the macular region near the Fuchs’ spot. Enlargement of the PPA was seen.

**Figure 3.**
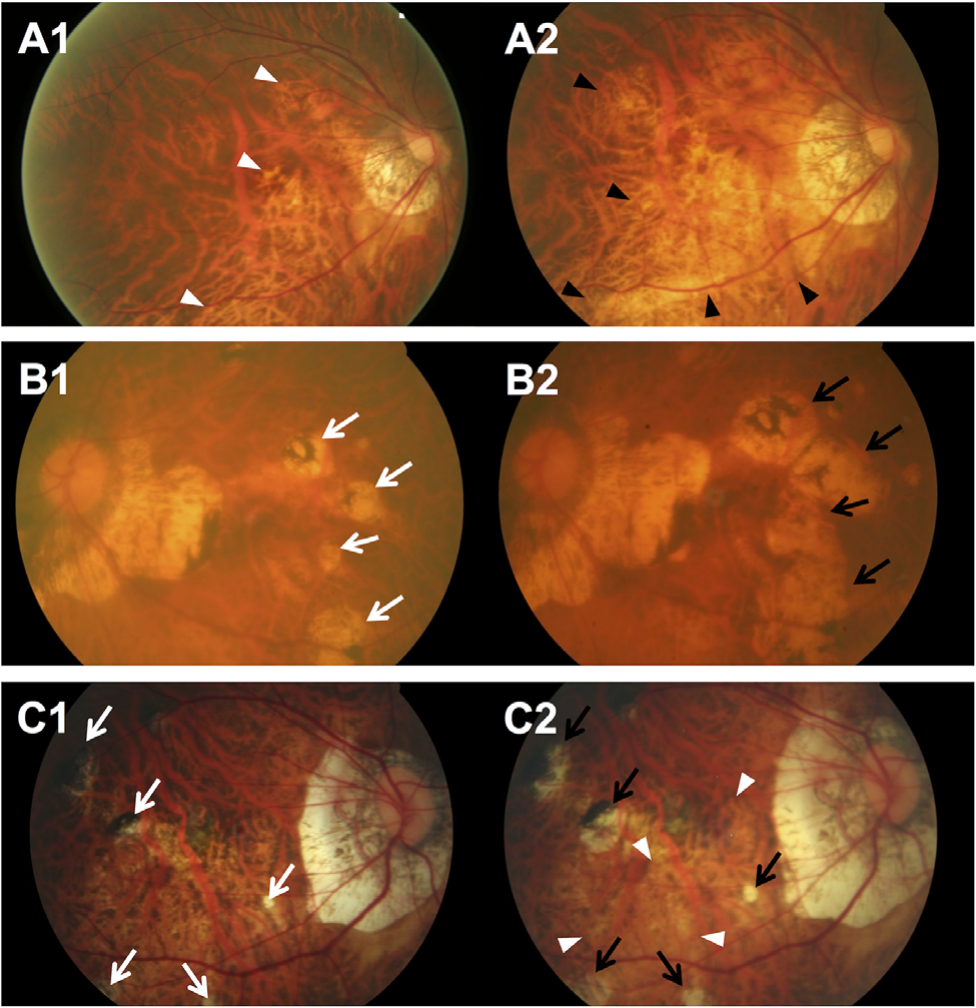
Fundus photographs of eyes showing MMD progression with enlargement of existing chorioretinal atrophy (no change in Meta-PM category) over a 6-year period. (**A**. Fundus photographs from a 60-year-old Malay woman showing progression of MMD (enlargement of diffuse chorioretinal atrophy) over a follow-up of 6 years. (**A1**) Image from the right eye obtained at baseline visit (SE of –5.6 D and AL of 28.8 mm) showing peripapillary diffuse chorioretinal atrophy (*white arrowheads*). (**A2**) Six years later, enlargement of diffuse chorioretinal atrophy (*black arrowheads*) across the macular region was seen, and enlargement of the PPA was observed. (**B**) Fundus photographs from a 73-year-old Malay woman showing progression of MMD (enlargement of patchy chorioretinal atrophy) over a follow-up of 6 years. (**B1**) Image from the left eye obtained at baseline visit (SE of –14.4 D and AL of 28.0 mm) showing several patchy chorioretinal atrophies temporal to macular (*white arrows*). (**B2**) Six years later, the fundus showed an enlargement of the patchy chorioretinal atrophy coalescing with other previous patchy chorioretinal atrophies (*black arrows*) toward the macula, but not involving macula. Enlargement of the PPA was seen as well. (**C**) Fundus photographs from a 45-year-old Chinese man showing progression of MMD (enlargement of both diffuse and patchy chorioretinal atrophy) over a follow-up of 6 years. (**C1**) Image from the right eye obtained at baseline visit (SE of –15.6 D and AL of 33.1 mm) showing multiple small pigmented patchy chorioretinal atrophies (*white arrows*) around the macula. (**C2**) Six years later, the fundus showed slight enlargement of the patchy chorioretinal atrophies (*black arrows*) around the macula and enlargement of the diffuse chorioretinal atrophy inferior to the macula (*white arrowheads*). Enlargement of the PPA was observed.

**Figure 4.**
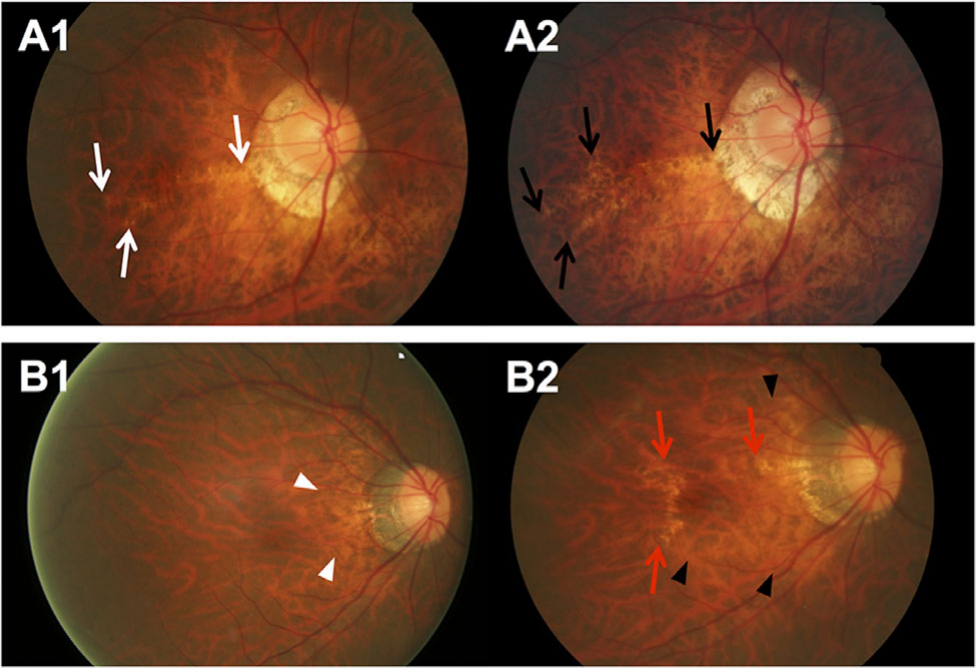
Fundus photographs of eyes showing MMD progression with changes in plus lesions over a 6-year period. (**A**) Fundus photographs from a 50-year-old Chinese man showing progression of MMD (widening of existing lacquer cracks) over a follow-up of 6 years. (**A1**) Image from the right eye obtained at baseline visit (SE of –14.0 D and AL of 30.0 mm) showing linear lacquer cracks across the macula (*white arrows*). (**A2**) Six years later, widening of existing lacquer cracks temporal to the macula was seen (*black arrows*). (**B**) Fundus photographs from a 47-year-old woman showing progression of MMD (enlargement of diffuse chorioretinal atrophy and new lacquer crack) over a follow-up of 6 years. (**B1**) Image from the right eye obtained at baseline visit (SE of –9.0 D and AL of 29.1 mm) showing slight peripapillary diffuse chorioretinal atrophy (*white arrowheads*). (**B2**) Six years later, enlargement of diffuse chorioretinal atrophy (*black arrowheads*) toward the macula and development of lacquer cracks (*red arrows*) superior and temporal to the macula were observed.

The 6-year cumulative progression of MMD was 17.0% (95% CI, 12.6%–21.4%) of 288 eyes with preexisting MMD in the SEED study. In the 49 eyes with MMD progression (Table 4), six (12.2%) had progression from Meta-PM category 2 to 3 with the development of new patchy chorioretinal atrophy (Fig. 2). Of 288 eyes with preexisting MMD, 33 eyes (11.5%) had enlargement of existing diffuse chorioretinal atrophy (Fig. 3A), five (1.7%) had enlargement of existing patchy chorioretinal atrophy (Fig. 3B), and two (0.7%) had enlargement of both existing diffuse and patchy chorioretinal atrophy (Fig. 3C) over the 6-year follow-up. For changes in plus lesions over the 6-year follow-up, four eyes (1.4%) had widening preexisting lacquer cracks and five eyes (1.7%) had development of new lacquer cracks (Fig. 4).

**Table 4.** Progression of MMD Lesions Over a 6-Year Period Among Eyes with Any MMD Progression in the SEED Study (*n* = 49 Eyes)

	Eyes With Any MMD
	Progression Over a
	6-Year Period
	(*n* = 49 Eyes)
Change in Meta-PM category	
Progression from Meta-PM category 2 to 3 (development of new patchy chorioretinal atrophy)	6 eyes (12.2)^*^
Progression from Meta-PM categories 2/3 to 4 (development of new macular atrophy)	0 eye (0.0)
Enlargement of existing chorioretinal atrophy	
Enlargement of existing diffuse chorioretinal atrophy	33 eyes (67.3)
Enlargement of existing patchy chorioretinal atrophy	7 eyes (14.3)^†^
Enlargement of existing macular atrophy	0 eye (0.0)
Changes in plus lesions	
Enlargement of existing lacquer crack(s)	4 eyes (8.2)^‡^
Existing MMD with new lacquer crack(s)	5 eyes (10.2)^§^
Existing MMD with new Fuchs’ spot/CNV	0 eye (0.0)

CNV, choroidal neovascularization.

* Of the 6 eyes with progression from meta-PM category 2 to 3 (development of new patchy chorioretinal atrophy), three had enlargement of existing diffuse chorioretinal atrophy.

† Of the seven eyes with enlargement of existing patchy chorioretinal atrophy, two had enlargement of existing diffuse chorioretinal atrophy.

‡ Of the four eyes with enlargement of existing lacquer crack(s), one had progression from meta-PM category 2 to 3 (development of new patchy chorioretinal atrophy).

§ Of the five eyes with new lacquer crack(s), one had enlargement of existing diffuse chorioretinal atrophy, and another had both enlargement of existing diffuse chorioretinal atrophy and progression from meta-PM category 2 to 3 (development of new patchy chorioretinal atrophy).

**Figure 5.**
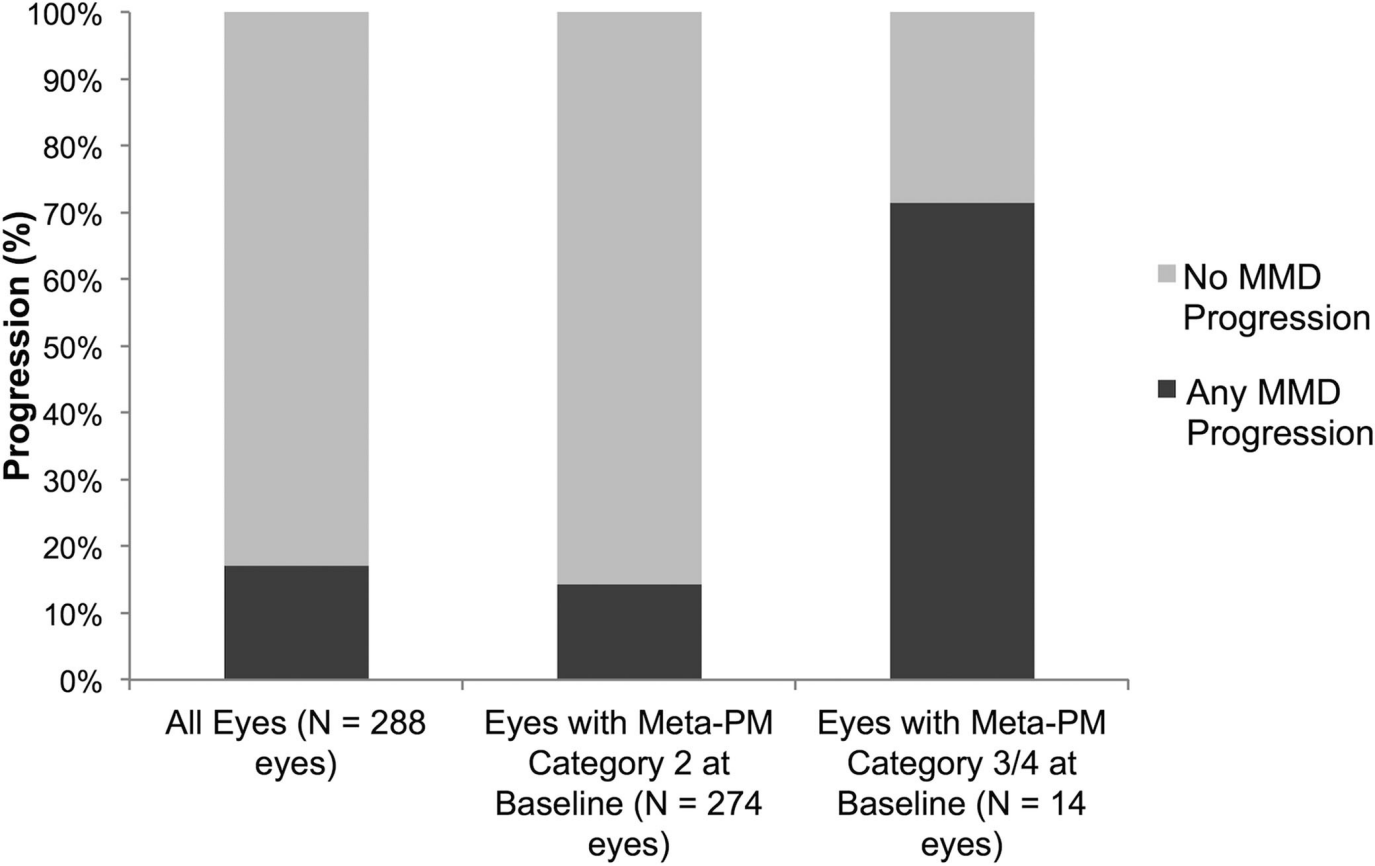
Progression of MMD lesions over a 6-Year period in myopic eyes with preexisting MMD in the SEED Study (*n* = 202 patients and 288 eyes) stratified by meta-PM categories at baseline examination.

The progression of MMD was higher at 71.4% in eyes with Meta-PM categories 3 or 4 at baseline than those with Meta-PM category 2 at 14.2% (*P* < 0.001; Fig. 5). In multivariable models, the risk of MMD progression increased significantly with severe MMD (Meta-PM categories 3 or 4) at baseline (RR, 2.5; *P* = 0.009), more myopic SE (RR of 1.1 per 1 D decrease; *P* = 0.02), and longer AL (RR of 1.3 per 1 mm increase; *P* = 0.02; Table 5). The progression of MMD was not significantly different between eyes with presence of cataract, glaucoma, AMD, and DR at baseline (all *P* > 0.05).

**Table 5.** The 6-Year Progression Rates of MMD Among Myopic Eyes With Preexisting MMD in the SEED Study (*n* = 202 Patients and 288 Eyes)

			Six-Year Eye-Specific		Unadjusted		Multivariate-Adjusted	
			Progression of MMD		RRs		RRs	
	No. at							
	risk	*n*	%	95% CI	RR (95% CI)	*P* Value	RR (95% CI)	*P* Value
Severity of MMD at baseline								
Meta-PM category 2	274	39	14.2	10.1–18.4	Reference		Reference	
Meta-PM categories 3 and 4	14	10	71.4	44.4–98.5	5.0 (3.1–8.2)	<0.001	2.5 (1.3–4.9)^*^	0.009
Age group at baseline, y								
40–49	42	11	26.2	12.3–40.1				
50–59	88	20	22.7	13.8–31.7				
60–69	88	10	11.4	4.6–18.1				
≥70	70	8	11.4	3.8–19.1				
*P* value for trend								
Age at baseline, per 1 y	288	49	–	–	1.0 (0.9–1.0)	0.02	1.0 (1.0–1.0)^*^	0.33
Ethnicity								
Non-Chinese	130	30	23.1	15.7–30.4	Reference		Reference	
Chinese	158	19	12.0	6.9–17.2	0.5 (0.3–1.0)	0.05	0.7 (0.3–1.4)^*^	0.31
Sex								
Male	141	20	14.2	8.4–20.0	Reference		Reference	
Female	147	29	19.7	13.2–26.2	1.4 (0.7–2.7)	0.32	1.2 (0.6–2.2)^*^	0.55
Myopia levels at baseline								
Low and moderate myopia (SE between –0.5 and –4.99 D)	144	15	10.4	5.4–15.5				
High myopia (SE ≤–5.0 D)	144	34	23.6	16.6–30.6				
SE at baseline, per 1 D	288	49	–	–	1.1 (1.1–1.2)	0.001	1.1 (1.0–1.1)^*^	0.02
AL at baseline, mm								
<26.5	152	15	9.9	5.1–14.7				
≥26.5	136	34	25.0	17.6–32.4				
AL at baseline, per 1 mm	288	49	–	–	1.3 (1.1–1.5)	0.001	1.3 (1.0–1.6)^†^	0.02
Education level								
Primary/lower education	130	28	21.5	14.4–28.7	0.6 (0.3–1.2)	0.14	0.6 (0.3–1.2)^*^	0.18
Secondary/above education	158	21	13.3	7.9–18.6	Reference		Reference	
Lacquer cracks at baseline								
Yes	18	7	38.9	13.9–63.8	2.5 (1.3–4.7)	0.005		
No	270	42	15.6	11.2–19.9	Reference			
Optic disc tilt at baseline								
Yes	159	35	22.0	15.5–28.5	2.0 (1.0–4.1)	0.05		
No	129	14	10.9	5.4–16.3	Reference			
Peripapillary ICC at baseline								
Yes	12	5	41.7	8.9–74.4	2.6 (1.1–6.0)	0.03		
No	274	44	16.1	11.7–20.4	Reference			
Cataract at baseline								
Yes	121	21	17.4	10.5–24.2	1.0 (0.6–1.9)	0.95		
No	159	27	17.0	11.1–22.9	Reference			

ICC, intrachoroidal cavitation.

* Multivariable modified Poisson regression models to obtain adjusted RRs were adjusted for severity of MMD at baseline, age, sex, ethnicity, education level, and SE and for correlation between eyes in each individual.

† Multivariable modified Poisson regression models to obtain adjusted RRs were adjusted for severity of MMD at baseline, age, sex, ethnicity, education level, and AL and for correlation between eyes in each individual.

### Associations of MMD With VA and VRQoL at the 6-Year Follow-up Visit

After excluding 664 participants with ocular comorbidities, 1493 participants without ocular comorbidities were included in the following analyses. Of these, 74 (5.0%) had presence of MMD at the 6-year follow-up visit.

The mean BCVA (in the better-seeing eye) of eyes with MMD was significantly worse (0.12; 95% CI, 0.10–0.14) than that of eyes without MMD (0.07; 95% CI, 0.07–0.07; *P* < 0.001), after multivariate adjustment (Table 6). The presence of any MMD was also independently associated with a significant decrement of 0.3 logits in the emotional domain of the IVI after adjusting for PVA, but not for the reading and mobility domains.

**Table 6.** BCVA and VRQoL Outcomes With MMD and Corresponding Meta-PM Categories at the 6-Year Follow-up in Adults Without Ocular Comorbidities^*^ in the SEED Study (*n* = 1493) Using Linear Regression Models

		BCVA in							
		Better-Seeing		Reading (IVI)		Mobility (IVI)		Emotional (IVI)	
		Eye (logMAR)		Score^†^		Score^†^		Score^†^	
	Number	Adjusted		Adjusted		Adjusted		Adjusted	
	of	mean		Mean		Mean		Mean	
	Eyes	(95% CI)^‡^	*P* Value	(95% CI)^§^	*P* Value	(95% CI)^§^	*P* Value	(95% CI)^§^	*P* Value
Any MMD									
No MMD	1419	0.07 (0.066–0.075)^‡^		6.78 (6.73–6.83)^§^	–	7.20 (7.15–7.25)^§^	–	6.61 (6.57–6.66)^§^	–
MMD	74	0.12 (0.10–0.14)^‡^	<0.001	6.63 (6.38–6.87)^§^	0.24	7.12 (6.89–7.36)^§^	0.54	6.32 (6.09–6.54)^§^	0.01
Severity of MMD									
No MMD	1419	0.07 (0.066–0.075)^‡^	–	6.78 (6.73–6.83)^§^	–	7.20 (7.15–7.25)^§^	–	6.61 (6.57–6.66)^§^	–
Meta-PM category 2	68	0.11 (0.08–0.13)^‡^	0.001	6.67 (6.42–6.92)^§^	0.41	7.18 (6.94–7.42)^§^	0.87	6.35 (6.12–6.58)^§^	0.03
Meta-PM categories 3 and 4	6	0.36 (0.28–0.43)^‡^	<0.001	5.85 (4.97–6.73)^§^	0.04	6.16 (5.32–7.00)^§^	0.02	5.75 (4.94–6.56)^§^	0.04

SD, standard deviation.

* Ocular comorbidities include cataract, glaucoma, AMD, and DR.

† For IVI, lower scores represent poorer reading, mobility, or emotional well-being.

‡ Adjustments for age, ethnicity, sex, education, and SE at the 6-year follow-up were made.

§ Adjustments for age, ethnicity, sex, education, SE, and PVA at the 6-year follow-up were made.

When we conducted analyses based on disease severity, eyes with Meta-PM categories 3 or 4 had significantly worse BCVA in the better-seeing eye (0.36; 95% CI, 0.28–0.43) than those with Meta-PM category 2 (0.11; 95% CI, 0.08–0.13; *P* = 0.001), after multivariate adjustment. The presence of Meta-PM categories 3 or 4 in the better-seeing eye was also independently associated with decrements of 0.9, 1.0, and 0.9 logits in reading (*P* = 0.04), mobility (*P* = 0.02), and emotional (*P* = 0.04) VRQoL scores after adjusting for PVA, respectively, compared with individuals with no MMD in the better-seeing eye.

### Incidence and Progression of MMD in Eyes With Previous Cataract Surgery

A total of 960 eyes with previous cataract surgery in 536 patients were analyzed, consisting of 851 eyes without MMD at baseline in 477 patients and 109 eyes with preexisting MMD in 65 patients.

**Figure 6.**
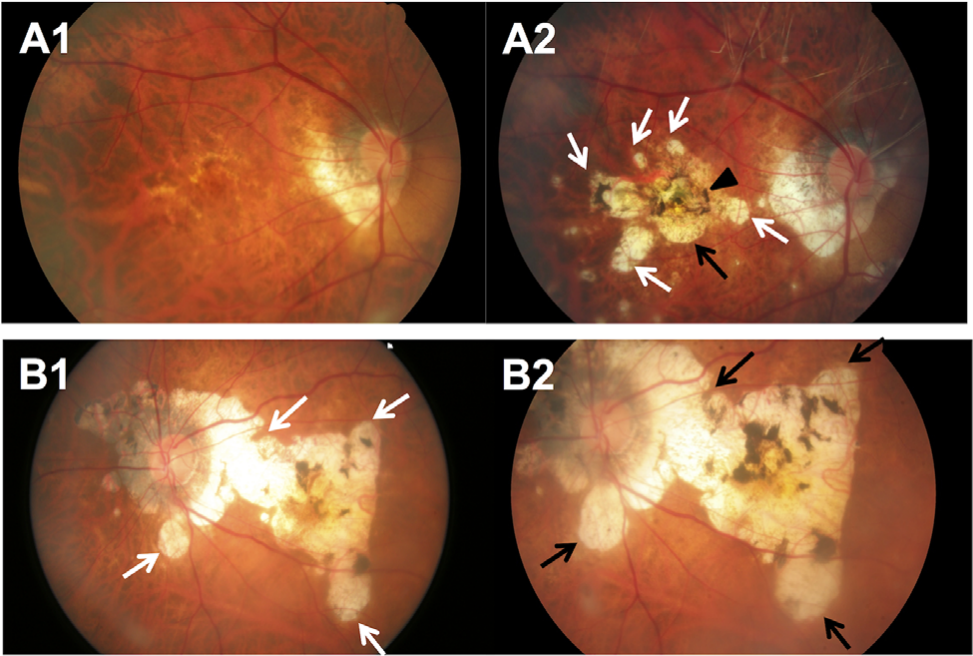
Fundus photographs of eyes with previous cataract surgery showing MMD progression over a 6-year period. (**A**) Fundus photographs from a 58-year-old Chinese man showing progression of MMD with development of new macular atrophy involving the fovea and new patchy chorioretinal atrophy over a follow-up of 6 years. (**A1**) Image from the right eye obtained at baseline visit (AL of 27.9 mm and with cataract surgery) showing diffuse chorioretinal atrophy in the macular region near the fovea. (**A2**) Six years later, macular atrophy (*black arrow*), several surrounding spotted patchy chorioretinal atrophies (*white arrows*), and choroidal neovascularization (*black arrowhead*) developed. (**B**) Fundus photographs from a 73-year-old Malay woman showing MMD progression (enlargement of both macular atrophy and patchy chorioretinal atrophy) over a follow-up of 6 years. (**B1**) Image from the left eye obtained at baseline visit (with cataract surgery) showing several patchy chorioretinal atrophies temporal to macular (*white arrows*). (**B2**) Six years later, the fundus showed an enlargement of the coalescence of macular atrophy and patchy chorioretinal atrophy (*black arrows*) from the macula with increased pigmentation.

The 6-year cumulative incidence of MMD was 0.4% (95% CI, 0.0%–0.8%) in 851 eyes with cataract surgery and no MMD at baseline in the SEED study. All of these eyes developed incident diffuse chorioretinal atrophy and had the presence of both tessellated fundus and PPA at baseline. The 6-year cumulative progression of MMD was 22.0% (95% CI, 14.1%–29.9%) of the 109 eyes with both preexisting MMD and cataract surgery. Of these 24 eyes (Table 7), 6 (25.0%) had an increase in Meta-PM categories, comprising 5 eyes with progression from Meta-PM category 2 to 3 (development of new patchy chorioretinal atrophy) and 1 eye with progression from Meta-PM category 2 to 4 (development of new macular atrophy; Fig. 6A). Enlargement of existing diffuse, patchy, and macular chorioretinal atrophy over the 6-year follow-up occurred in five (4.6%), nine (8.3%), and four (3.7%) eyes, respectively (Fig. 6B). For changes in plus lesions, one (0.9%), one (0.9%), and two (1.8%) eyes had development of new lacquer cracks, choroidal neovascularization, and Fuchs’ spot, respectively.

**Table 7.** Progression of MMD Lesions Over a 6-Year Period Among Eyes with Both MMD Progression and Cataract Surgery in the SEED Study

	Eyes With Both MMD
	Progression Over a
	6-Year Period
	and Baseline
	Cataract Surgery
	(*n* = 24 eyes)
Change in Meta-PM category	
Progression from Meta-PM category 2 to 3 (development of new patchy chorioretinal atrophy)	5 eyes (20.8)
Progression from Meta-PM category 2 to 4 (development of new macular atrophy)	1 eye (4.2)^*^
Progression from Meta-PM category 3 to 4 (development of new macular atrophy)	0 eye (0.0)
Enlargement of existing chorioretinal atrophy	
Enlargement of existing diffuse chorioretinal atrophy	5 eyes (20.8)
Enlargement of existing patchy chorioretinal atrophy	9 eyes (37.5)
Enlargement of existing macular atrophy	4 eyes (16.7)^†^
Changes in plus lesions	
Existing MMD with new lacquer crack(s)	1 eye (4.2)^‡^
Existing MMD with new Fuchs’ spot/CNV	3 eyes (12.5)

CNV, choroidal neovascularization.

* Of the eye with progression from Meta-PM category 2 to 4 (development of new macular atrophy), the same eye had development of new patchy chorioretinal atrophy, new Fuchs’ spot, and new CNV.

† Of the four eyes with enlargement of existing macular atrophy, one had enlargement of existing patchy chorioretinal atrophy and two had new Fuchs’ spots.

‡ Of the eye with new lacquer crack(s), the same eye had both progression from Meta-PM category 2 to 3 (development of new patchy chorioretinal atrophy) and expansion of existing diffuse chorioretinal atrophy.

## Discussion

In the 6-year follow-up period, the incidence and progression of MMD was low at 1.2% and 17.0%, respectively, in the SEED study population of Asian myopic adults aged 40 years or older. For the new MMD cases, the majority (95%) developed diffuse chorioretinal atrophy. For eyes with MMD progression, the most frequent change was enlargement of existing diffuse chorioretinal atrophy (71.4%), followed by enlargement of existing patchy chorioretinal atrophy (14.3%), and new patchy chorioretinal atrophy with progression from Meta-PM category 2 to 3 (12.2%). Eyes with severe MMD are at greater risk of progression than those with mild MMD. The negative impact of severe MMD on vision and several VRQoL parameters presents a potential public health problem.

### Incidence of MMD

We found a 6-year eye-specific cumulative incidence of MMD of 1.2% among myopic eyes of adults aged 40 years or older in the SEED study, which was higher than that of the Handan Eye Study (0.22% over a 5-year period among 2236 myopic eyes of adults aged ≥30 years residing in rural China).^15^ Similar to our findings, the development of diffuse chorioretinal atrophy (Meta-PM category 2) was predominant in new MMD cases in the Handan Eye Study.^15^ Our higher incidence of MMD could be related to the higher prevalence of myopia and high myopia (35.8% and 6.4%, respectively) in our population, compared with the latter study (22.8% and 1.4%, respectively).^15^ However, the disparity in incidence among populations may also be due to varying age compositions in each study population.^14^ In contrast with the relatively high prevalence of MMD in Singapore, the incidence of MMD illustrates slow degenerative changes in MMD development. The MMD prevalence rate was determined by cumulative MMD cases over many years of myopia, but our reported incidence rate referred to a short duration of 6 years only. The development of MMD is a slow process that may require a long duration of myopia. Our findings of the association between MMD development and age support this, as older age may be a surrogate for a longer duration of myopia.

### Risk Factors for MMD Development

Consistent with previous cross-sectional population-based studies^6^^–^^10^ and clinic-based studies on MMD,^17^^,^^18^ we found that older age, greater myopic SE, and longer AL were associated with MMD development. Fundus tessellation at baseline was present for all the newly developed MMD cases in our study, which is aligned with Hayashi's hypothesis on fundus tessellation as a first marker for MMD development.^18^ The presence of fundus tessellation is associated with thinner choroidal thickness,^36^^–^^39^ and the latter presents morphologic changes that increases the risk of development of chorioretinal atrophy lesions.^40^^,^^41^

### Progression of MMD

Our finding of the 6-year cumulative progression of MMD at 17.0% among 288 eyes with existing MMD in the SEED study was similar to that of the Beijing Eye Study (5-year progression of 15.1%; *n* = 139 eyes; patients aged ≥40 years) and the Blue Mountains Eye Study (5-year progression of 23.9%; *n* = 46 eyes; patients aged ≥49 years), but lower than that of the Handan Eye Study (5-year progression of 35.3%; *n* = 51 eyes; patients aged ≥30 years).^6^^,^^7^^,^^15^ The differences in progression rates of MMD among these populations may be due to varying definitions of MMD adopted (e.g., Curtin's classification), different age compositions, different myopia severity levels, and various ethnicities in each study population. Despite the varying factors in each population, a relatively similar progression rate of MMD (15%–25%) was reported in most studies.^18^

Similar to our study, the progression pattern of MMD showed that enlargement of existing chorioretinal atrophy and development of new chorioretinal atrophy occurred most frequently in previous studies in Australia and China over a 5-year follow-up period.^6^^,^^7^^,^^15^ Similar progression patterns of MMD were observed in clinic-based retrospective case series studies in Japan.^17^^,^^18^ However, a higher frequency of occurrence of advanced MMD progression patterns was observed, such as development of macular atrophy and choroidal neovascularization. This difference may be due to the longer follow-up periods of more than 10 years and these patients who tend to have severe MMD are not representative of the general population.

### Risk Factors for MMD Progression

Consistent with previous studies, progression of MMD was associated with worse SE^15^^,^^16^ and longer AL at baseline^16^^–^^18^ in our study. More severe myopia levels are associated with longer axial elongation, which may result in predisposition of the fundus to atrophic changes.^42^^,^^43^

We found that eyes with severe MMD (Meta-PM categories 3 or 4) at baseline are at greater risk of MMD progression than eyes with mild MMD (Meta-PM category 2) after multivariate adjustment. Compared with eyes with mild MMD, eyes with severe MMD tend to have worse myopic SE and longer AL, which may present thinner chorioretinal layers in the posterior pole that predisposes the eye to higher risk of MMD progression.^3^^,^^44^ Closer monitoring and management of patients with severe MMD are needed as these patients who already have more severe disease status are likely to deteriorate even further.

We found no association between MMD progression and age, which contrasts with some studies that reported increased risk of MMD progression with older age,^16^^,^^17^ but were consistent with others, such as the Beijing Eye Study and Handan Eye Study.^7^^,^^15^ The lack of association between MMD progression and age may be due to the small number of patients with MMD progression and short follow-up period of 5 to 6 years compared with studies that found a significant relationship, which had longer follow-up periods of at least 10 years.^16^^,^^17^

### Cataract Surgery Patients

The risk of MMD development and progression in aphakic and pseudophakic persons has not been documented in current literature. Furthermore, some studies on MMD excluded those with previous cataract surgery, because the SE values do not accurately reflect myopia status and severity.^7^^,^^10^^,^^18^ Persons with cataract and previous cataract surgery tend to be older and more myopic,^45^^–^^47^ which is associated with a higher risk of MMD. In our study, the 6-year incidence of MMD was 0.4% and the progression of MMD was 22.0% in eyes with previous cataract surgery. Pseudophakic eyes had a lower incidence of MMD, but higher progression of MMD than phakic eyes. This finding may possibly be explained by the older age of pseudophakic patients, because the risks of both cataract and MMD increase with older age. Because we have excluded cataract surgery patients who had a higher progression of MMD, the progression of MMD in our study population is likely to be underestimated.

### Impact of MMD on VRQoL

We found that eyes with severe MMD (Meta-PM categories 3 or 4) had poorer vision and experienced a significant decrement in the reading, mobility, and emotional domains of VRQoL that was independent of VA. However, this may be due to other factors, such as a decrease in contrast sensitivity, depth perception, and peripheral vision, which may affect visual function in those with MMD.^48^ As such, larger studies are needed to elucidate the comprehensive impact of MMD on the visual functioning system, as well as visual rehabilitative strategies to optimize reading abilities, promote mobility, and provide counselling to improve VRQoL and mental health in individuals with severe MMD.

The strengths of this study included a large, population-based, longitudinal design with a reasonable follow-up rate (76%), and the use of standardized and comprehensive methodologies to assess study exposures and outcomes at both visits. Limitations include a loss to follow-up of 30% resulting in a potential selection bias and an underestimation of the incidence and progression of MMD. The 6-year MMD incidence (1.2%) and progression (17.0%) rates reported from the combined SEED study population may not be fully representative of the general population in Singapore, because there are slight variations in MMD incidence and progression rates among the Chinese, Malay, and Indian participants. However, the incidence and progression rates reported from the combined SEED study population provide an approximation of the rates in Singapore, a multiethnic urban Asian city. We excluded participants with previous cataract surgery from the main analyses,^11^ which is likely to have resulted in an underestimation of the progression of MMD in our population, owing to the exclusion of a greater proportion of myopic participants with past cataract surgery who are likely to have MMD.^45^^–^^47^ Posterior staphyloma is part of the definition of pathologic myopia, but it was not investigated because wide-field imaging systems were not available in this study at follow-up. Subtle changes in lacquer cracks might be missed in fundus photographs without the use of fluorescein angiography or indocyanine green angiography.

To conclude, in contrast with the relatively high prevalence of MMD in Singapore, the 6-year cumulative incidence (1.2%) and progression (17.0%) of MMD illustrates slow degenerative changes in MMD. If an individual does not have MMD in adulthood, the risk of developing MMD is low. However, if an individual already has signs of MMD, the risk of progression of MMD is relatively high. Moreover, eyes with severe MMD are at greater risk of progression than those with mild MMD. These findings highlight the need for closer monitoring and management of patients with severe grades of MMD, which are associated with poorer BCVA and VRQoL outcomes. Worse SE and longer AL were strong predictors of both MMD development and progression, underlining the need for myopia control interventions in early life,^49^ which may then reduce the risk of MMD development and progression in adulthood.^50^
